# Sustainable Valorization of Rice Straw into Biochar and Carbon Dots Using a Novel One-Pot Approach for Dual Applications in Detection and Removal of Lead Ions

**DOI:** 10.3390/nano15010066

**Published:** 2025-01-03

**Authors:** Jagpreet Singh, Monika Bhattu, Meenakshi Verma, Mikhael Bechelany, Satinder Kaur Brar, Rajendrasinh Jadeja

**Affiliations:** 1 Faculty of Engineering & Technology, Marwadi University, Rajkot-Morbi Road, Rajkot 360003, Gujarat, India; jagpreetnano@gmail.com; 2 Department of Chemistry, Research and Incubation Centre, Rayat Bahra University, Mohali 140103, Punjab, India; 3 Centre of Research Impact and Outcome, Chitkara University, Rajpura 140417, Punjab, India; 4 Department of Applied Science, Chandigarh Engineering College, Chandigarh Group of Colleges Jhanjeri, Mohali 140307, Punjab, India; 5 Institut Européen des Membranes (IEM), UMR-5635, University of Montpellier, ENSCM, CNRS, Place Eugène Bataillon, CEDEX 5, 34095 Montpellier, France; 6 Functional Materials Group, Gulf University for Science and Technology (GUST), Mubarak Al-Abdullah 32093, Kuwait; 7 Department of Civil Engineering, Lassonde School of Engineering, York University, Toronto, ON M3J 1P3, Canada

**Keywords:** adsorption, biochar, carbon dots, environmental remediation, fluorescence sensor, lead, rice straw, SDGs, waste water treatment

## Abstract

Lead (Pb) is a highly toxic heavy metal that causes significant health hazards and environmental damage. Thus, the detection and removal of Pb^2+^ ions in freshwater sources are imperative for safeguarding public health and the environment. Moreover, the transformation of single resources into multiple high-value products is vital for achieving sustainable development goals (SDGs). In this regard, the present work focused on the preparation of two efficient materials, i.e., biochar (R-BC) and carbon dots (R-CDs) from a single resource (rice straw), via a novel approach by using extraction and hydrothermal process. The various microscopic and spectroscopy techniques confirmed the formation of porous structure and spherical morphology of R-BC and R-CDs, respectively. FTIR analysis confirmed the presence of hydroxyl (–OH), carboxyl (–COO) and amine (N–H) groups on the R-CDs’ surface. The obtained blue luminescent R-CDs were employed as chemosensors for the detection of Pb^2+^ ions. The sensor exhibited a strong linear correlation over a concentration range of 1 µM to 100 µM, with a limit of detection (LOD) of 0.11 µM. Furthermore, the BET analysis of R-BC indicated a surface area of 1.71 m^2^/g and a monolayer volume of 0.0081 cm^3^/g, supporting its adsorption potential for Pb^2+^. The R-BC showed excellent removal efficiency of 77.61%. The adsorption process followed the Langmuir isotherm model and second-order kinetics. Therefore, the dual use of rice straw-derived provides a cost-effective, environmentally friendly solution for Pb^2+^ detection and remediation to accomplish the SDGs.

## 1. Introduction

The rapid increase in industrialization and population has led to continuous environmental depletion due to heavy metal contamination, which poses significant environmental challenges. The growth in anthropogenic activities, such as metal-based surface plating, mining, and the large-scale production of paper, steel, fertilizers, pesticides, and metal-based batteries, has significantly contributed to the increase of toxic inorganic contaminants in water bodies. This issue is particularly pronounced in developing regions, where industrial practices are often less regulated [1]. Nowadays, there has been a growing need to adopt highly efficient and sustainable techniques for the detection of heavy metals [2]. Heavy metals such as copper, lead, mercury, and chromium are extremely poisonous and pose serious threats to both humans as well as to the environment, even when present in trace amounts [3,4,5,6]. Moreover, consuming food and water contaminated by industrial metal effluent causes major health problems such as kidney, liver, central nervous system, bone, cancer, and tooth damage and may even lead to death [7,8]. Among the various heavy metal ions, lead (Pb^2+^) is considered the most toxic due to the associated health risks, including developmental delays in children, kidney dysfunction, hemolytic anemia, increased blood pressure, and neurodegenerative diseases such as Alzheimer’s and Parkinson’s. Chronic exposure to lead is also linked to hypertension, reproductive toxicity, and cognitive impairments [9]. Thus, reliable sensing and elimination methods are essential for accurately analyzing and sequestrating heavy metal levels in different sources to effectively control environmental pollution [10,11].

The growing concern regarding the toxicity of Pb^2+^ underscores the urgent need for reliable methods to sense and remediate these contaminants to ensure safe drinking and irrigation water. To date, there are various analytical techniques, including mass spectrometry, resonance light scattering method [12], X-ray fluorescence analysis [13,14,15,16,17], inductively coupled atomic emission spectrometry, UV–Vis spectrophotometric [18], and fluorescence spectroscopy, ref. [19] have been extensively utilized for the detection of Pb^2+^ in aqueous matrices. Additionally, conventional remediation methods like membrane separation, catalysis, chemical precipitation, adsorption, oxidation/reduction, and biological treatment are also employed for the removal of Pb^2+^ from wastewater [20]. Undoubtedly, these techniques possess high sensitivity and reliability. However, the performance might be hindered by various limitations like costliness, complexity, and lengthy procedures, limiting their widespread application. Therefore, there is a need for much simpler, more sensitive, faster, and cost-effective detection methods for remediating Pb^2+^ [21,22,23].

Along with water contamination, waste management is also considered the most pressing environmental challenge faced by modern society. In agriculture-dominant countries like India, this issue is particularly pronounced due to the substantial generation of crop residues and the lack of efficient management practices. The most common and expedient method for disposing of these biomass residues, particularly rice and wheat straw, is open burning, which is widely adopted due to its low cost and ease of implementation. However, this practice not only contributes to air pollution but also contributes to environmental degradation and poses significant risks to the public.

Therefore, addressing these challenges necessitates innovative approaches that align with the principles of circular economy and sustainability. For this purpose, the most effective strategy is the utilization of waste materials to create multiple valuable products, thereby minimizing waste and reducing the environmental footprint.

The conversion of agricultural waste residues into nanomaterials (NMs) and carbon-based materials with remarkable physicochemical properties has positioned them as promising candidates for various environmental applications [23,24]. Since their discovery in 2004, carbon dots (CDs) have emerged as a notable addition to the family of carbon nanomaterials. CDs have several advantages over conventional fluorescent dyes and semiconductor quantum dots, including cost-effectiveness, excellent water solubility, non-toxicity, and multicolor fluorescence. These attributes have garnered significant interest in CDs for diverse applications such as light-emitting diodes, polymerization, ionic detection, sensing, cell labeling, and photocatalysis [25]. Various synthesis techniques and precursors have been explored for CD synthesis, with an emphasis on cost-effective, environmentally friendly biomass sources [26]. Numerous biomass precursors have been studied for their potential in CD synthesis, including turtle shells [27], crop residues [28], straws, agricultural waste [29], gram shells [30], and mushrooms [31]. In addition to CDs, the preparation of biochar from agricultural waste is widely regarded as an eco-friendly approach for waste management and water remediation.

Considering the remarkable potential of CDs and BC, we have developed two efficient materials—biochar (R-BC) and carbon dots (R-CDs)—from a single agricultural waste resource (rice straw) using a novel green synthesis approach. This is the first time such a method has been applied to rice straw, demonstrating its potential for waste valorization and environment remediation. The R-CDs were found to be highly effective as lead (Pb) sensors, showing excellent sensitivity and selectivity in detecting lead ions in contaminated water. Furthermore, the R-BC produced from the same rice straw was successfully utilized for the removal of lead from aqueous solutions, offering an environmentally friendly and cost-effective solution for water purification. By producing multiple functional materials from a single waste resource, this approach not only reduces waste but also contributes to sustainable water management. The dual application of R-CDs as sensors and R-BC as adsorbents highlights the potential of rice straw as a valuable resource in environmental remediation efforts. Along with the dual applicability, cost-effectiveness is one of the most prominent factors, as rice straw is a cheap and plentiful agricultural residue that is being utilized as the single raw material for the synthesis of both R-BC and R-CDs. Moreover, in terms of reliability and efficiency, the study exhibits excellent consistency and stability in the detection of Pb^2+^ ions, with R-CDs showing a low detection limit of 0.11 µM and a broad linear range, which surpasses the sensitivity of many existing sensors for Pb^2+^ detection. Overall, this study underscores the importance of innovative green technologies in tackling the pressing challenges of heavy metal pollution and waste management.

## 2. Materials and Methods

### 2.1. Chemicals Reagents

The study employed analytical reagent (AR) grade materials, including metal salts *viz* lead nitrate, barium nitrate, ferric nitrate, ferrous nitrate, manganese nitrate, magnesium nitrate, sodium nitrate, chromium nitrate, copper nitrate, cobalt nitrate (90–98% purity, obtained from Sigma Aldrich, St. Louis, MO, USA). These materials were utilized without undergoing further purification procedures. Water samples were taken from the Majha region of Punjab in India. Deionized (DI) water was used as the solvent for solution preparation.

### 2.2. Synthesis of R-CDs

**Figure 1:**
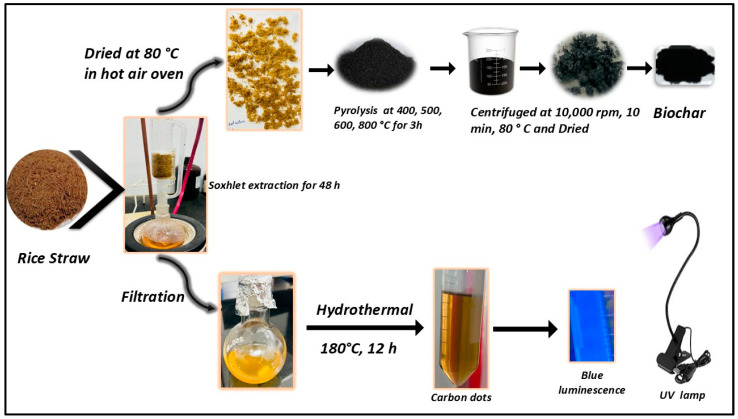
Stepwise process of biochar and carbon dots synthesis.

For the preparation of R-CDs, initially, the rice straw was thoroughly rinsed with tap water and then washed with distilled water. Afterward, the washed straw was dried in a hot air oven at 100 °C for 12 h and then ground into fine powder. A 5 g sample of powdered rice straw was subjected to Soxhlet extraction using water as the solvent for a duration of 48 h. The extended Soxhlet extraction process ensures the thorough recovery of phytochemicals from the precursor biomass. These phytochemicals, including phenolic compounds, organic acids, and flavonoids, are instrumental in forming functional groups such as hydroxyl (–OH), carboxyl (–COOH), and carbonyl (–C=O) on the biochar surface during pyrolysis. The presence of these functional groups enhances the material’s surface reactivity, facilitating interactions with target contaminants through mechanisms such as electrostatic attraction, hydrogen bonding, and metal-ligand coordination. The resulting extract was filtered, and 30 mL of the extract was transferred into a Teflon-lined autoclave. Initially, the extract was placed in the muffle furnace at varying temperature conditions, including 180 °C for 6 h, 12 h, and 18 h. Upon completion of the reaction, the mixture was allowed to cool to room temperature. The resulting solution was then centrifuged at 10,000 rpm for 15 min, followed by three subsequent washing with deionized water (Figure 1). The supernatant containing the R-CDs was subsequently analyzed and confirmed using UV-visible and fluorescence spectroscopy. Among all the optimization conditions for the R-CDs preparation, the best results were obtained at 180 °C for 12 h.

### 2.3. Preparation of R-BC

The residue obtained in the extraction process was further utilized for the R-BC preparation. The collected residue was dried at 80 °C for 24 h and then transferred to a crucible for further hydrothermal treatment. The R-BC is prepared at varying temperature conditions such as 400 °C, 500 °C, 600 °C, and 800 °C. The prepared biochar was further mixed in 50 mL of deionized water and subjected to sonication for half an hour. The suspension was then centrifuged at 10,000 rpm, with three sequential rinsing, each lasting 10 min. The remaining residue was again dried in a hot air oven for 2 h (Figure 1). The maximum yield of R-BC is obtained at 600 °C for 3 h, which is calculated using the below equation.

$$Biochar\;yield\left(\%\right)=(\frac{Mass\;of\;Biochar\;(g)}{Mass\;of\;rawmaterial\;(g)})\;*\;100$$

The yield of the obtained R-BC was calculated to be 44.29%.

### 2.4. Characterization Techniques

The prepared R-CDs and R-BC were characterized using high-resolution transmission electron microscopy (HRTEM, JEOL-2100 Plus HRTEM, Tokyo, Japan) images and energy-dispersive X-ray spectroscopy (EDS). Furthermore, the images were analyzed using ImageJ (IJ 1.53k) to find out the average size of R-CDs. Fourier transform infrared (FTIR) spectra were analyzed using a PerkinElmer FTIR spectrophotometer, Shelton, CT, USA to monitor the presence of functional groups on R-CDs. Scanning electron microscopy (SEM), X-ray Diffraction (XRD), and Brauner Emmett Teller (BET) studies were conducted to analyze the surface morphology, surface area, and porosity of prepared biochar.

### 2.5. Photophysical Studies

The prepared R-CDs were screened against different heavy metals using photoluminescence spectroscopy. However, there is no significant change has been observed in the photoluminescence intensity of R-CDs with heavy metals except Pb^2+^. This signifies that the prepared R-CDs showed selectivity towards Pb^2+^. Therefore, further studies, such as titration and interference studies, were conducted to evaluate the sensitivity and selectivity of prepared R-CDs toward Pb^2+^.

### 2.6. Lead Ion (Pb^2+^) Adsorption Study

Pb^2+^ removal was conducted by preparing a stock solution with a known concentration of Pb^2+^ (100 ppm). This stock solution was diluted using DI water to make various concentrations varying from 5 to 20 ppm for experimental analysis. A calibration curve was generated by measuring concentrations between 5–20 ppm using spectroscopic analysis at λ_max_ = 290 nm.

The adsorption capacity q_t_ (mg/g) was measured as given below:

(1) $$q_{t}=\frac{\left(C_{i}-{\;C}_{t}\right)}{m}\times V$$

where, C_i_ is the initial concentration of Pb^2+^, C_t_ is the concentration at time t. m (g) is the weight of R-BC taken, and V (L) is the volume of Pb^2+^solution taken. Moreover, the percentage efficiency was measured by using the below equation (Equation (2)) [32]

(2) $$\%\;dye\;removal=\frac{C_{i}-{\;C}_{t}}{C_{i}}\times 100$$

## 3. Results

This section has been divided into four sub-sections related to characterization, sensing, and adsorption studies of the prepared P-CDs and R-BC.

### 3.1. Characterization of R-BC

#### 3.1.1. Scanning Electron Microscopy Analysis

**Figure 2:**
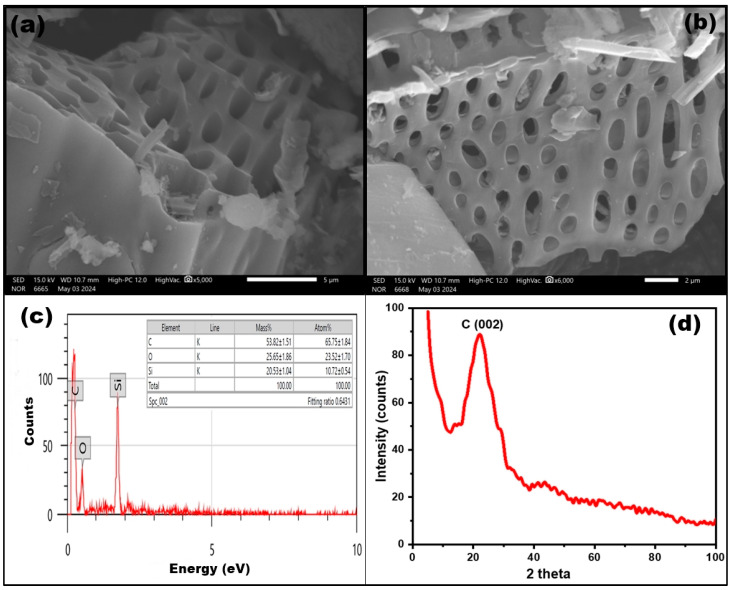
Morphological and elemental analysis of Biochar: (**a**,**b**) SEM images, (**c**) EDX analysis spectrum, (**d**) XRD pattern.

The surface morphology of R-BC was analyzed using SEM (Figure 2a,b). The SEM images reveal isolated, thin cylindrical structures with a high density of pores. The surface of the R-BC was observed to be rough with repetitive, empty pore channels. The expansion of pore widths indicates structural evolution, where the BC matrix retains its rigidity, and the pore walls remain interconnected. These features suggest the presence of mesopores and macropores within the R-BC structure. The EDX spectra confirmed the presence of a larger extent of carbon in the prepared sample (Figure 2c).

#### 3.1.2. XRD Analysis

The phase structure of R-BC was analyzed using XRD over a 2θ range of 10° to 80°. As shown in Figure 2d, the XRD pattern exhibits a broad peak around 2θ~22°, which corresponds to graphite carbon diffraction along the (002) planes. The extracted biochar showed a great variation from the XRD pattern of biochar derived from rice straw, as no additional peaks for silica were observed. This variation is attributed to the removal of volatile components during the extraction process, which leads to a reduction in the silica content and an increase in carbonaceous matter. The XRD data confirmed the highly amorphous nature of the synthesized biochar.

#### 3.1.3. Brunauer–Emmett–Teller Analysis

**Figure 3:**
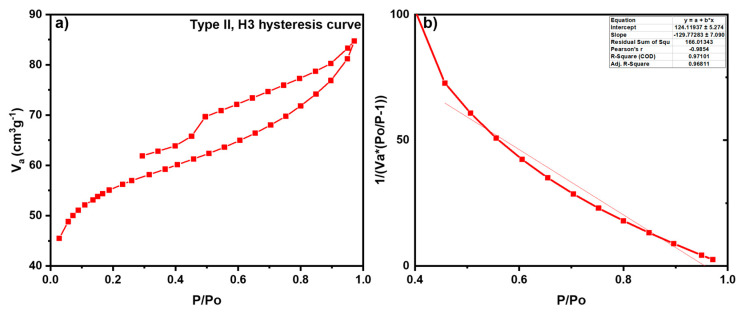
(**a**) N_2_ adsorption–desorption isotherm of biochar; (**b**) BET curve illustrating the surface area and monolayer volume of biochar.

**Figure 4:**
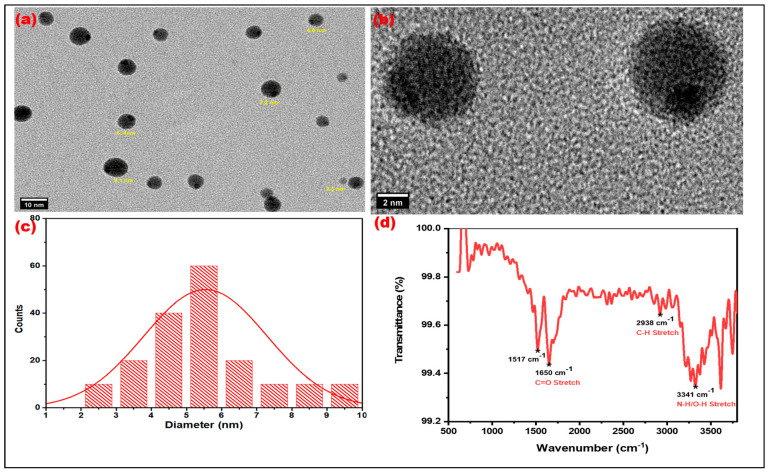
Microscopic and surface chemistry analysis of R-CDs: (**a**,**b**) TEM images of R-CDs illustrating the spherical-shaped R-CDs, (**c**) Size distribution histogram illustrating the average size of R-CDs from 8–10 nm, and (**d**) FTIR spectra to explore the surface functionality.

The N_2_ adsorption and desorption analysis was performed to evaluate the porosity of the prepared biochar (Figure 3a). The N_2_ adsorption at STP follows Type II isotherm with H3 hysteresis loop, which suggests the simultaneous presence of meso and macropores in biochar, and the desorption closes abruptly due to the cavitation phenomenon. Furthermore, the BET analysis was performed using the equation,

$$\frac{1}{V_{a}(\frac{P_{o}}{P}-1)}=\frac{C-1}{V_{m}C}\left(\frac{P}{P_{o}}\right)+\frac{1}{V_{m}C}$$

where, V_a_ = Volume of gas adsorbed; V_o_ = saturation vapor pressure; V_m_ = monolayer volume; C = BET constant. The plot of P/P_o_ against 1/Va (P_o_/P−1) gives a linear curve (Figure 4b), and the monolayer volume obtained was found to be 0.0081 cm^3^/g. Moreover, the surface area of biochar was observed to be 1.71 m^2^/g.

### 3.2. Characterization of R-CDs

#### 3.2.1. HRTEM Analysis

HRTEM analysis showed the presence of quasi-spherical particles with monodisperse structures with a diameter of 8–10 nm (Figure 4a–c). This narrow size range is significant as it suggests good control over the synthesis process, resulting in consistent particle sizes that can lead to improved properties for various applications, including sensing and remediation. Additionally, the analysis illustrates the amorphous nature of R-CDs. The amorphous nature of the R-CDs may contribute to their versatility in various applications, as they can offer advantages in terms of surface functionalization and chemical reactivity compared to crystalline counterparts. Additionally, the lack of crystallinity is attributed to enhanced fluorescence characteristics [33].

#### 3.2.2. FTIR Spectroscopic Studies

FTIR analysis indicated the presence of various kinds of functional groups on R-CDs’ surface (Figure 4d). The broad transmittance band at 3611 and 3341 cm^−1^ corresponds to hydroxyl (–OH) groups and amine (N–H) groups, respectively, suggesting a high degree of hydrophilicity, which enhances their solubility in aqueous solutions. Additionally, the strong peaks observed at 1517 cm⁻^1^ and 1650 cm⁻^1^ indicate an increased presence of carboxylic functional groups [34,35].

#### 3.2.3. Optical Characterization of R-CDs

**Figure 5:**
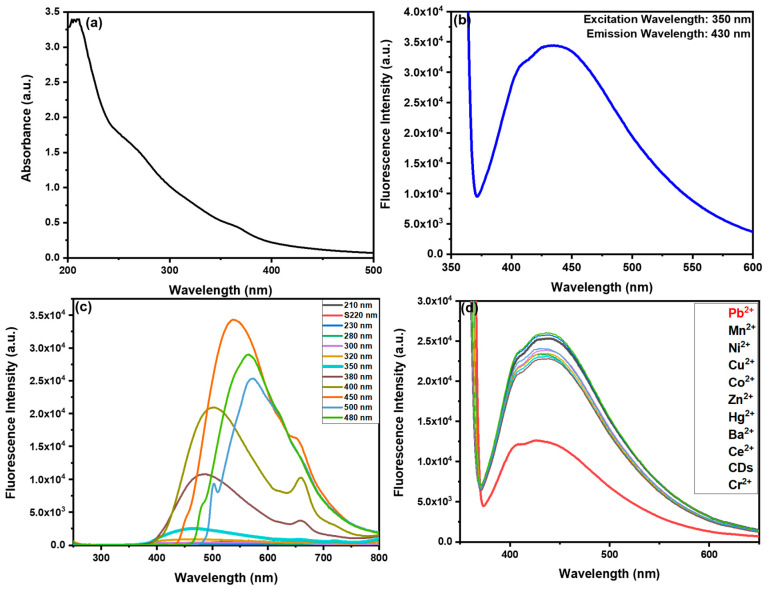
(**a**) Absorption spectra of R-CDs; (**b**) fluorescence spectra of synthesized R-CDs exhibiting an emission band at 430 nm; (**c**) Excitation-dependent emissive fluorescence profile of R-CDs exhibiting a red shift in λ_em_; (**d**) Screening of R-CDs against various heavy metals illustrating a high quench in the presence of Pb^2+^ and the other metal ions do not exhibit any effect on the fluorescence behaviour of CDs.

The optical characteristics were investigated using UV–visible (UV–Vis) and photoluminescence (PL) spectroscopy. The R-CDs exhibited distinctive absorbance spectra, featuring a prominent peak at 220 nm corresponding to π–π* transition (Figure 5a) [36]. R-CDs displayed negligible fluorescence under daylight conditions, whereas in the presence of UV light, CDs exhibit blue fluorescence with an emission peak at 430 nm (at λ_ext_. = 350 nm), consistent with characteristic emission profiles of CDs (Figure 5b). The synthesized R-CDs showed excitation-dependent fluorescence, a characteristic feature in carbon-based nanomaterials [37]. As depicted in Figure 5c, varying the excitation wavelength from 220 nm to 380 nm resulted in a progressive red shift of the fluorescence emission peak, along with variations in emission intensity. These shifts likely arise from differences in particle size distribution or diverse surface energy traps. As the size of CDs varies from 8–10 nm, thus quantum confinement effect is less pronounced in these dots as compared to smaller CDs. Even within the 8–10 nm range, slight variations in particle size can cause differences in electronic bandgap energies. This results in photons of different wavelengths being absorbed and emitted, leading to excitation-dependent PL [38,39]. Along with the energy band gaps, ensemble effects play a significant role. Although single CDs can exhibit excitation-dependent PL, in bulk samples, the size and surface variability across the ensemble lead to overlapping emissions from different subsets of particles [40].

### 3.3. Sensing Study of Pb^2+^

The emission spectra of the R-CDs were analyzed at different concentrations, revealing that lower concentrations resulted in higher sensitivity and maximum emission. Additionally, the stability of the R-CD’s fluorescence was evaluated, which is a critical attribute for sensing applications. The R-CDs demonstrated remarkable colloidal stability, showing no visible precipitation or agglomeration even after one month of storage. Notably, the fluorescence intensity remained virtually unchanged over this period, indicating the R-CDs possess outstanding long-term fluorescent stability, making them promising candidates for sensor development. The synthesized R-CDs were screened for their sensing response against various heavy metal ions using a fluorescence spectrophotometer with λ _ex_ = 350 nm. For this purpose, 60 µL of metal nitrate solution was introduced into 3 mL of a 10% diluted R-CDs solution. Among the metal ions tested, no significant change in the fluorescence emission of the R-CDs was observed, except in the case of Pb^2+^, which caused a quenching of the fluorescence intensity (Figure 5d).

**Figure 6:**
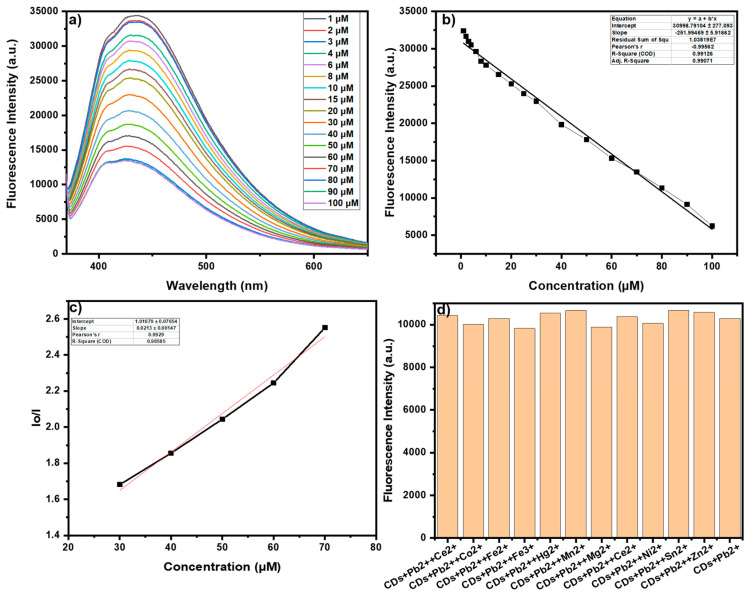
(**a**) Declination in fluorescence spectra of R-CDs on titration with Pb^2+^ (1 µM–100 µM); (**b**) Linear decreasing response of R-CDs towards the subsequential increase in the Pb^2+^ over 1 µM–100 µM; (**c**) Stern Volmer Plot of R-CDs towards the detection of Pb^2+^; (**d**) Interference studies of other potential competing ions for R-CDs towards Pb^2+^.

The inhibition of fluorescence intensity might be attributed to the formation of metal (Pb^2+^) complexes with the surface functional groups of R-CDs. Furthermore, to assess the sensitivity of prepared R-CDs towards Pb^2+^, titration studies were conducted by subsequently adding the target analyte (Pb^2+^) solution to the diluted R-CDs solution ranging from 1 µM to 100 µM (Figure 6a). An increase in Pb^2+^ concentration led to a decrease in the emission spectra of R-CDs, which suggests that the fluorescent probe exhibits a linear response over a concentration range of 1 µM to 100 µM (Figure 6b).

Furthermore, the sensitivity and quenching efficiency of synthesized R-CDs were evaluated using a Stern–Volmer (SV) plot. By observing λ_em_ = 430 nm, a relationship was plotted (Stern–Volmer plot), representing the normalized inhibition of luminescent behavior in fluorescence intensity of the R-CDs with increasing concentrations of Pb^2+^ (1 µM to 100 µM), which was employed to plot SV and calculate the quenching constant by applying the SV Equation (3):

I_o_/I_i_ = 1 + K_sv_[Q] (3)

where I_o_ and I_i_ present fluorescence intensities of unquenched R-CDs and on the addition of varying concentrations of quencher, [Pb^2+^]. The parameters [Q] and K_sv_ denote the quencher concentration and the SV constant, respectively. The SV equation was employed to determine the quenching constant. A plot of I_o_/I_i_ versus the quencher concentration [Q] was generated (Figure 6c). The quenching constant was measured to be 2.14 × 10^4^ M^−1^. For sensitivity analysis, the limit of detection (LOD) and the limit of quantification (LOQ) were estimated using the 3σ method. Equations (4) and (5) [19].

LOD = 3.3σ/s(4)

LOQ = 10σ/s(5)

In the above equation,

‘σ’ is the standard deviation,

‘s’ is related to the slope of the calibration plot.

The LOD and LOQ for Pb^2+^ ion were estimated to be 0.11 µM and 0.34 µM, respectively, over a linear range from 1 to 100 µM. The LOD was found to be below permissible limits set by regulatory agencies such as the WHO (10 µg/L or ~0.048 µM for drinking water). Thus, this approach is well-suited for on-site monitoring and practical applications in environmental remediation and water quality management. Table 1 illustrates the detection efficacy of various kinds already reported CDs in literature.

For the component to be a good sensor, it must be selective toward the target analyte in the presence of other potential interfering components. Hence, interference studies have been conducted in which 60 µL of metal nitrate solution (100 µM) was added to the solution of R-CDs and Pb^2+^ (3 mL of diluted R-CDs (10%) + 60 µL Pb^2+^). Interestingly, the other metal ions do not show any effect on the emissive response of R-CDs with Pb^2+^ (Figure 6d). All the abovementioned studies indicate that the fluorescent probe is highly selective and sensitive towards the detection of Pb^2+^.

Moreover, Pb^2+^ ion detection requires the effect of various important factors including pH, temperature, and the incubation time. In this regard, the fluorescence stability of R-CDs and R-CDs with Pb^2+^ was evaluated due to the reduced protonation of R-CDs. Intriguingly, the luminescent intensity of R-CDs exhibited negligible variation in alkaline conditions (pH 8–14). Furthermore, the fluorescence response of R-CDs and complex remained stable even under fluctuating temperature conditions (25–40 °C). Additionally, the synthesized R-CDs-based probes exhibited exceptional photostability evidenced by the consistent photoluminescence intensity observed even after prolonged excitation with a UV lamp. For the stability check, the R-CDs were kept for a time of two months, and interestingly, no precipitation (cm^−1^) or floating particles were found, and no considerable variation in the fluorescence response was observed, which signifies the higher stability of R-CDs.

Moreover, the relative quantum yield was measured by considering fluorescein as reference material as per the below equation:

$$\varphi_{A}=\varphi_{S}\;*\;(F_{A}/F_{S})\;*\;({O.D.}_{S}/{O.D.}_{A})$$

where φ is the quantum yield; F is the relative emission; O.D. is the absorbance respectively; and the subscript “A” and “S” stands for the sample and standard. Thus, using the aforementioned parameters, the fluorescence yield was calculated to be 13%.

#### Mechanism Illustrated for Pb^2+^ Recognition Using R-CDs

**Figure 7:**
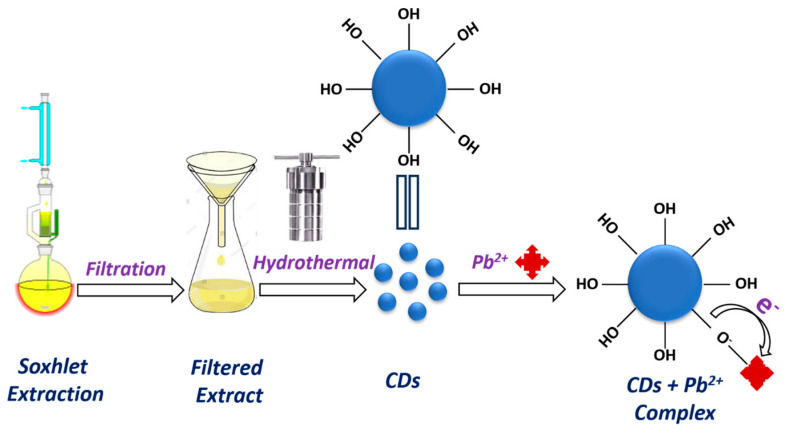
Illustration of the chelation between R-CDs and Pb^2+^ via the formation of coordination bonds between the lone pairs present on the surface functional groups with the electron-deficient Pb^2+^.

The inhibition in the fluorescence response of R-CDs by the addition of Pb^2+^ is primarily attributed to the chelation between Pb^2+^ and –OH functionalities of R-CDs (Figure 6). This chelation results in an electron transfer process and promotes the non-radiative recombination of excitons, which results in a notable reduction in fluorescence intensity [41,42,43,44]. The selective detection is attributed to the strong affinity and rapid chelation of Pb^2+^ with –OH functionalities compared to other metal ions (Figure 7). The selective binding of Pb^2+^ towards CDs is owing to the soft Lewis acidic nature of metal and offers high affinity for electron-rich sites, especially by –OH groups. Additionally, the relatively large ionic radius and high polarizability of Pb^2+^ enhance its interaction with the –OH groups compared to smaller, harder metal ions like Na^+^ or Mg^2^⁺. The underlying mechanism involves the formation of a coordination bond between the lone pairs on the oxygen atoms of the –OH groups and the empty orbitals of Pb^2+^. This bond formation facilitates an efficient electron transfer from the R-CDs to the Pb^2+^ ions, which competes with the radiative recombination process responsible for fluorescence. Consequently, exciton recombination becomes predominantly non-radiative, leading to fluorescence quenching. The high stability of the Pb^2+^–R-CDs complex is crucial in this context, as it ensures a reliable and reproducible quenching response, thereby enhancing the selectivity and sensitivity of the detection system.

**Table 1:** Comparative analysis of recently reported techniques for Pb^2+^.

Techniques	Detection Range (ng/mL)	Detection Limit (ng/mL)	References
Pb^2+^-driven DNA molecular device	0.02–1 µM	20 nM	[45]
AGRO100	0–1000 nM	1.0 nM	[46]
G-quadruplex DNAzyme	0–1000 nM	0.4 nM	[46]
Blue and Red CDs	Not Given	2.89 nM	[47]
Fluorescent starch-based hydrogel	5–160 μg/L	0.06 μg/L	[48]
*Moringa oleifera* gum derived CDs	0–100 ppb	11.62 nM	[49]
**Rice straw derived CDs**	**1–100 µM**	**0.11 µM**	**This Work**

### 3.4. Point of Zero Charge (pH_zpc_)

The point of zero charge (pH_zpc_) of the adsorbents was determined using the salt addition method to evaluate the surface characteristics of the adsorbents and the role of pH in the adsorption process. In this regard, 0.01 M KNO_3_ solution was used in the studies to measure the pH_zpc_. For this, 0.1 M HCl or 0.1 M NaOH was used to bring the solution’s starting pH (pH 0) down to values between 2 and 10. In an Erlenmeyer flask, 100 mL of the pH-adjusted solution was mixed with a determined quantity of biosorbent (20 mg). To guarantee equilibrium, the mixture was shaken with a magnetic shaker for 48 h at room temperature. A plot of pH_*f*_ vs. pH_0_ was created after the concentration of the supernatant was measured. The intersection of the curve with the line pH_*f*_ = pH_0_ was determined to be the point of zero charge.

**Figure 8:**
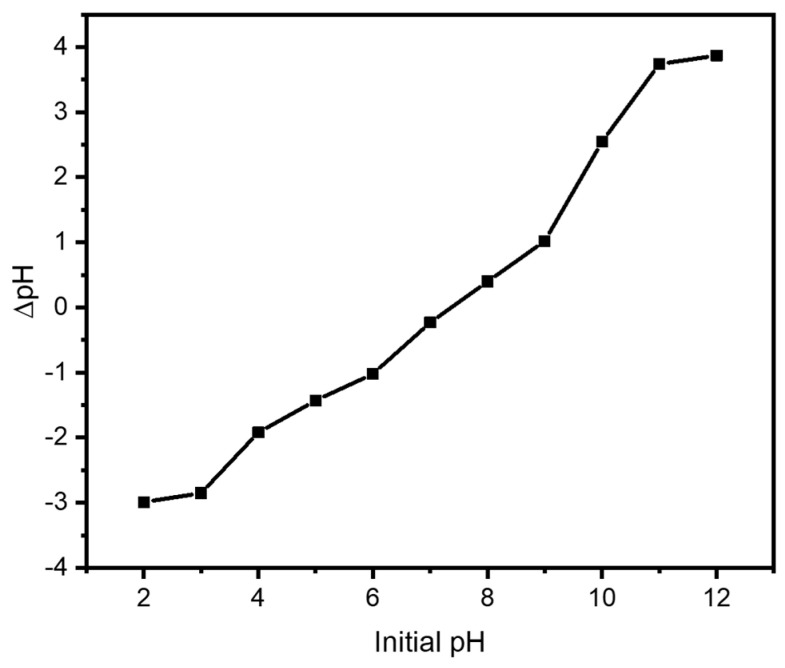
Point of zero charge study for R-BC.

The pH_zpc_ values for the Pb–R-BC were found to be 7.8. The increased concentration of H^+^ ions at low pH reduces the adsorption effectiveness by competing with Pb^2+^ ions for the active adsorption sites. The H^+^ ion concentration falls with increasing pH, which causes the functional groups on the adsorbent surface to deprotonate and increases the number of binding sites that are available. Because of the improved interaction with negatively charged adsorbent surfaces and less electrostatic repulsion, this promotes the adsorption of Pb^2+^ ions. Protonation causes the adsorbent surface to become positively charged at pH values lower than pH_zpc_, which promotes the adsorption of anionic species. On the other hand, the surface gains a negative charge at pH values higher than pH_zpc_, which improves the adsorption of cationic Pb^2+^ ions (Figure 8) [50].

### 3.5. Lead Removal Studies

**Figure 9:**
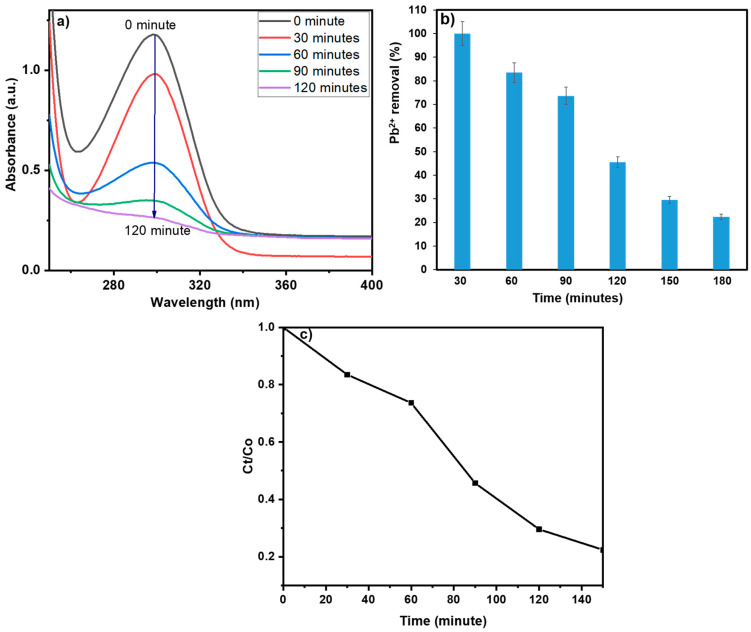
(**a**) Illustration of decrease in Pb^2+^ concentration with time on the addition of biochar-BC; (**b**,**c**) Trend in Pb^2+^ removal efficiency with time.

The prepared biochar exhibits high potential for the removal of the Pb^2+^ owing to the abundance of oxygen and nitrogen-containing surface functionalities. Pb^2+^ ions display a maximum absorbance at λ_max_ = 290 nm, and the addition of R-BC results in a significant decrease in absorbance over time. The adsorption process reaches equilibrium after 150 min, with no further decrease in absorbance observed beyond this point. The biochar demonstrates a removal efficiency of 77.61%, corresponding to an adsorption capacity of 155.234 mg/g (Figure 9a). This efficiency is largely due to the biochar’s surface charge properties, which promote electrostatic interactions with Pb^2+^ ions, facilitating effective sequestration (Figure 9b,c).

**Figure 10:**
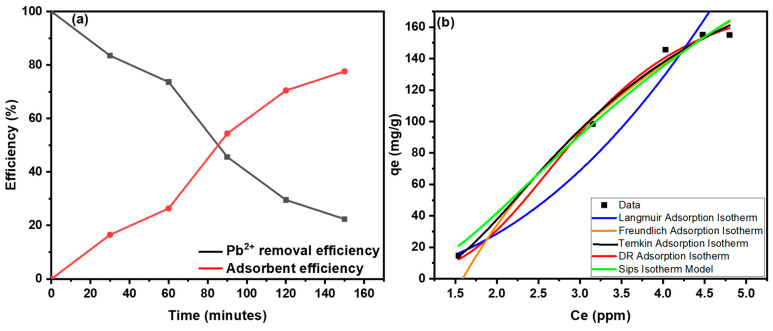
(**a**) Illustration of decrease in Pb^2+^ concentration with time on the addition of R-BC; (**b**) Graphical representation for Langmuir adsorption isotherm, Freundlich adsorption isotherm, Temkin isotherm model, DR Adsorption model, and Sips isotherm model.

Furthermore, the variables, including R-BC dosage, temperature, and concentration of analyte, were optimized, and optimal conditions were achieved with 10 mg of R-BC in 100 mL of a 20-ppm lead solution, and the pH is maintained as depicted by the pH_zpc_ study. The time course study depicts the rate of removal of Pb^2+^ and the rate of increasing adsorbent efficiency which are inversely proportional to each other (Figure 10a). A positive linear correlation has been observed between R-BC dosage and Pb^2+^ elimination efficiency though a saturation point is reached beyond which increasing the biochar amount has no further impact on removal efficiency. These results were observed due to the occupancy of available adsorption sites on the R-BC surface, limiting further lead ion uptake [51].

Moreover, the adsorption equilibrium data for the removal of Pb^2+^ ions were analyzed using the Langmuir, Freundlich, Temkin, Dubinin–Radushkevich, and Sips isotherm models to evaluate the relationship between the metal adsorption capacity (Q) and the equilibrium metal ion concentration (C_eq_) [32]. The Langmuir model illustrates monolayer adsorption on a homogeneous surface, whereas the Freundlich model accounts for adsorption on heterogeneous surfaces and the formation of multilayers [52]. However, the Temkin model applies to systems where adsorbate–adsorbent interactions are minimal [53]. The adsorption parameters for Pb (II) derived from these models are summarized in Table 2.

The maximum adsorption capacity (Q_m_) for a complete monolayer, as estimated by the Langmuir equation, cannot be determined directly using the Freundlich model. The model suitability was evaluated based on determination coefficients (R^2^) and the consistency of equilibrium constants and maximum adsorption capacities with experimental data. The Langmuir, Freundlich, Temkin, Dubinin–Radushkevich and Sips fits are graphically depicted in Figure 10b. Further, the Dubinin–Radushkevich isotherm model was fitted, which suggests that the physical adsorption occurs in the adsorption of Pb^2+^ using R-BC via weak Vander Waal forces. Additionally, the Sips model combines elements of both the Langmuir and Freundlich isotherms and is applicable for localized adsorption without interactions between adsorbed molecules. At low adsorbate concentrations (CeC_eCe), the isotherm simplifies the Freundlich model. Conversely, at higher concentrations, it reflects the Langmuir model, predicting a maximum monolayer adsorption capacity. The general expression for the SIPS isotherm is given in Table 2, which illustrates a very low R^2^ value, i.e., 0.62.

Among all the fits, the Langmuir isotherm model yielded a maximum adsorption capacity (q_max_) of 183 mg/g with the highest R^2^ value, demonstrating the high affinity of R-BC for Pb^2+^ ions. The Langmuir constant (K_L_ = 0.01295 L/mg) further supports the material’s strong adsorption potential, reflecting efficient monolayer adsorption on homogenous active sites. These findings align with the structural and surface properties of R-BC, including its functional groups and porosity, which facilitate selective Pb^2+^ ion binding.

The Langmuir model yielded the highest correlation coefficient (R^2^), indicating its suitability in describing Pb^2+^ adsorption on R-BC, with a theoretical monolayer capacity (Q_m_) of 183.8 mg/g, closely matching the experimental value of 152.5 mg/g. This suggests a strong agreement between the experimental data and the Langmuir model, underscoring the biochar’s high potential as an adsorbent compared to other agricultural waste-derived materials (Table 3).

### 3.6. Kinetics Studies

The kinetics of Pb^2+^ onto R-BC were studied to understand the adsorption mechanism, optimized removal conditions, and assess the rate of Pb^2+^ uptake. Various kinetic models, including pseudo-first-order (PFOM), pseudo-second-order (PSOM), Elovich model and Intraparticle diffusion model, were employed.

#### 3.6.1. PFOM

This model considers that during the initial phase of adsorption, the uptake of Pb^2+^ is directly proportional to the saturation concentration and adsorbent capacity [54]. It is typically observed when adsorption occurs through interfacial diffusion. The model is defined by the equation shown in Table 4.

**Figure 11:**
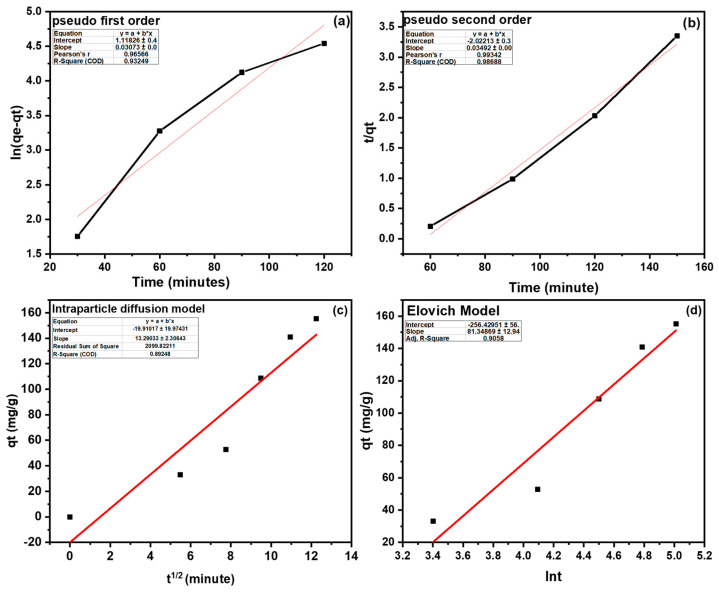
Graphical representation for PFOM (**a**), PSOM (**b**), Intraparticle diffusion model (**c**), and Elovich model (**d**).

A linear plot of log(q_e_ − q_t_) versus time for Pb^2+^ adsorption is used to determine the rate constants, as shown in Figure 11a. The kinetic constants are summarized in Table 4. The high correlation coefficient (R^2^ = 0.93) indicates that the adsorption process follows PFOM. Similarly, both the modeling of Pb^2+^ adsorption onto R-BC often show rapid uptake in the initial stages, followed by equilibrium being reached after several hours.

#### 3.6.2. PSOM

The model considers chemisorption as the rate-limiting step, where adsorption occurs through chemical interactions rather than solely through physical processes such as diffusion [55]. In this model, the adsorption rate depends on the availability of active adsorption sites rather than the concentration of adsorbate, making it more suitable for predicting adsorption behavior over the entire process [56]. The PSOM equation is shown in Table 4.

A plot of t/q_t_ versus time (t) provides the correlation, as shown in Figure 11b, allowing for the determination of *k*_2_ and *q*_e_ with the relevant parameters summarized in Table 5.

This model is often preferred over the pseudo-first-order model due to its better accuracy in predicting the equilibrium adsorption capacity and higher correlation coefficient (R^2^ = 0.98). In studies of Pb^2+^ removal using biochar, the PSOM has been shown to more accurately define the adsorption kinetics, indicating that chemisorption plays a dominant role in the process, similar to findings in other adsorbent systems [57].

#### 3.6.3. Intraparticle Diffusion Model

The intraparticle diffusion model (IDM) is employed to elucidate the adsorption mechanism of Pb^2+^. This model establishes a relationship between adsorption equilibrium and the adsorption uptake as a function of the square root of time (t^1/2^), providing insights into the diffusion-controlled processes governing the adsorption behavior (Table 4, Figure 11c).

#### 3.6.4. Elovich Model

The Elovich model (EKM) is widely employed to describe adsorption processes and is well-suited for adsorption occurring on heterogeneous surfaces. It is often utilized to analyze second-order kinetic processes, assuming a high degree of heterogeneity at the solid adsorbent interfaces. The general form of the Elovich equation, as given in Table 4, provides a framework for understanding the adsorption dynamics under these conditions. The plot of lnt against qt was utilized to calculate the constants as described in Table 5 (Figure 11d).

Among the models fit, the R^2^ values for the models (PFOM and PSOM) are quite close (0.93 and 0.98). Hence, MRD (%) was calculated for both the models using the equation below and the values were found to be 9.4% and 22.3% for PFOM and PSOM, respectively. Hence, the model with a lower MRD% value, i.e., PFOM, shows better agreement with the experimental results.

To further analyze the adsorption kinetics and evaluate the role of diffusion during the adsorption process, the intraparticle diffusion model was applied (Table 4). For the BC0, the model exhibited a moderate fit with an R^2^ value of 0.89, suggesting that lead (Pb^2+^) adsorption involved multiple mechanisms beyond simple diffusion. While the diffusion of Pb^2+^ ions from the bulk solution to the external surface of the biochar played a significant role, the rate-limiting step could be attributed to intraparticle diffusion into the pores. These findings indicate that while intraparticle diffusion contributes to the overall adsorption process, its extent is strongly influenced by the structural characteristics of the biochar, including pore size distribution and surface modifications.

## 4. Real Sample Analysis

The synthesized R-CDs exhibited notable sensitivity and specificity towards Pb^2+^ ions. To assess the practical utility and viability of the R-CDs, three water samples were employed using control and spiked particulars, in which a Pb^2+^ solution of different concentrations was spiked in the collected samples. The spiked samples were found to show a recovery varying from 96 to 101%, and the detailed recovery analysis is given in the table. The sample analysis suggests that CDs exhibit potential fluorescent probes for Pb^2+^ recognition (Table 6).

## 5. Conclusions and Future Perspectives

In this study, R-CDs and R-BC were successfully synthesized using a hydrothermal method involving rice straw as a precursor. TEM and BET studies confirmed that the prepared R-BC showed a porous structure and followed Type II isotherm with an H3 hysteresis loop, which confirmed the presence of meso and macropores. HRTEM analysis showed the presence of quasi-spherical particles with a diameter of 8–10 nm. The R-CDs exhibited a blue emission spectrum around λ_em_ = 430 nm at λ_ex_ = 350 nm. The resulting R-CDs showed remarkable sensing potential towards Pb^2+^ ions with an LOD of 0.11 µM. Furthermore, the R-BC showed a Pb^2+^ removal efficiency of 77.61%, with the adsorption process fitting the Langmuir isotherm and following second-order kinetics. These results demonstrate that the dual use of rice straw-derived materials offers a cost-effective, sustainable alternative for the detection and remediation of environmental pollutants. The developed method demonstrated a practical applicability by validated real water samples which gives satisfactory spiked recoveries.

However, one of the primary challenges is the relatively low surface area of R-BC, which might limit its adsorption capacity, particularly at higher pollutant concentrations. Thus, there is an imperative need for further optimization of synthesis methods to enhance the surface area and pore structure of R-BC for improved adsorption performance. Future studies should focus on optimizing the synthesis process to enhance the surface area and functionalization of R-BC for broader pollutant removal capabilities. Incorporating these materials into advanced systems, such as integrated remediation platforms or microfluidic devices, could significantly improve their practical application.

## Figures and Tables

**Table 2:** Different isotherm models for adsorbent.

Sr. No.	Models	Equations	Parameters
1.	Langmuir	$q_{e}=\frac{q_{m}\;*\;K_{L}\;*\;C_{e}}{1+K_{L}\;*\;C_{e}}$	q_e_ = amount of adsorbate per mass of adsorbent in equilibrium (mg/g), q_m_ = adsorption capacity in monolayer (mg/g) K_L_ = constant of the Langmuir model related to the binding energy.
2.	Freundlich	qe=Kf ∗ Ce1n	K_f_ = Freundlich capacity constant 1/n = Freundlich intensity
3.	Temkin Isotherm Model	$q_{e}=\frac{RT}{b_{T}}\ln\left(K_{T}\;*\;C_{e}\right)$	T = reaction temperature in Kelvin (K), R = gas constant b = Temkin constant
4.	Dubinin–Radushkevich Isotherm Model	$q_{e}=q_{m}{exp}^{-K(RTln(1+\left(1+\left(\frac{1}{C_{e}}\right)\right){)}^{2}}$	T = reaction temperature (K) R = gas constant K = D–R constant q_m_ = maximal adsorption capacity
5.	Sips Isotherm model	$q_{e}=q_{m}({\left(K\;*\;C_{e}\right)}^{n})/(\;{\left(1+K\;*\;C_{e}\right)}^{n})$	K = Sips Constant

**Table 3:** Determination of constant values for isotherms.

Sr. No.	Isotherms	Determination Constants			
1.	Langmuir	q_m_	K_L_	R_L_	R^2^
		183	0.01295	0.794281176	0.9909
2.	Freundlich	1/n	K_f_		R^2^
		2.16	6.41		0.81
3.	Temkin	BT	AT		R^2^
		147.82	0.63		0.9
4.	Dubinin–Radushkevich	q_m_	K_DR_		R^2^
		241.6	11.31		0.98
5.	Sips model	q_m_	K_s_		R^2^
		440.28	24.09		0.94

**Table 4:** Different Kinetic models for adsorbent.

Sr. No.	Models	Equations	Parameters
1.	PFOM	$log\left(q_{e}-q_{t}\right)=log\left(q_{e}\right)-\frac{k_{1}}{2.303}t$	q_e_ (mg/g) and q_t_ (mg/g) = quantity of Pb^2+^ adsorbed initially and at time t; k_1_ (L/min) = PFO rate constant.
2.	PSOM	$\frac{t}{q_{t}}=\frac{1}{k_{2}q_{e}^{2}}+\frac{t}{q_{e}}$	*q*_e_ (mg/g) = quantity of Pb^2+^ adsorbed at equilibrium; *k*_2_ (g/mgmin) = PSO rate constant.
3.	Intraparticle diffusion model	$q_{t}=K_{ip}t^{0.5}+C$	K_ip_ = intraparticle diffusion apparent adsorption rate constant C = intraparticle diffusion model constant
4.	Elovich model	$q_{t}=\frac{1}{b}(\ln\left(t\right)+\ln\left(ab\right))$	a = primary rate of adsorption b = desorption constant during each experiment

**Table 5:** Correlation constants for kinetic models.

Sr. No.	Kinetics Model	Parameters		
1.	PFOM	K_1_ (gmg^−1^ min^−1^)	R^2^	MRD (%)
		0.0257	0.93	7.57
2.	PSOM	K_2_ (gmg^−1^ min^−1^)	R^2^	MRD (%)
		0.000047	0.98	2.77
3.	Intraparticle diffusion model	K_ip_	R^2^	MRD (%)
		13.29	0.89	27.23
4.	Elovich model	b	R^2^	MRD (%)
		0.012	0.90	21.53

**Table 6:** Real sample analysis using the synthesized sensor.

Real Sample	Concentration of Pb^2+^ (µM)	Recovery of Pb^2+^ (µM)	Recovery of Pb^2+^ (%)
River Water	0.11	0.107	97.27
	1	0.98	98
	10	9.8	98
Tap Water	0.11	0.105	95.45
	1	0.96	96
	10	9.7	97
RO Water	0.11	0.107	97.27
	1	0.99	99
	10	9.753	97.53

## Data Availability

Data will be available on request.
